# Essential Oils May Lead *α*-Synuclein towards Toxic Fibrils Formation

**DOI:** 10.1155/2016/6219249

**Published:** 2016-05-24

**Authors:** Dina Morshedi, Mahour Nasouti

**Affiliations:** National Institute of Genetic Engineering and Biotechnology, Department of Industrial & Environmental Biotechnology, Shahrak-e Pajoohesh, km 15, Tehran-Karaj Highway, P.O. Box 14965/161, Tehran, Iran

## Abstract

*α*-Synuclein (*α*-Syn) fibrillation links with Parkinson's disease (PD) and several related syndromes. It is believed that exposure to the factors which promote fibrillation may induce and progress such neurodegenerative diseases (NDs). Herein, the effects of some wildly used essential oils including* Myrtus communis* (*M. communis*) on *α*-Syn fibrillation were examined.* M. communis* particularly increased *α*-Syn fibrillation in a concentration dependent manner. Given that applications of* M. communis* are very extensive in Asian societies, especially Zoroastrians, this study was extended towards its role on *α*-Syn fibrillation/cytotoxicity. By using a unilamellar vesicle, it was shown that the aggregated species with tendency to perturb membrane were increased in the presence of* M. communis*. In this regard, the cytotoxicity of *α*-Syn on SH-SH5Y cells was also increased significantly. Inappropriately, the effects of fibrillation inhibitors, baicalein and cuminaldehyde, were modulated in the presence of* M. communis*. However, major components of* M. communis* did not induce fibrillation and also the effect of* M. communis* was limited on other fibrinogenic proteins. Assuming that essential oils have the ability to pass through the blood brain barrier (BBB) along with the popular attention on aromatherapy for the incurable ND, these findings suggest an implementation of fibrillation tests for essential oils.

## 1. Introduction

Alterations in the structure of proteins associated with the formation of amyloid fibrils lead to several NDs. Synucleinopathies are progressive neurodegenerative disorders characterized by the accumulation of impact protaneous structures with high content of *α*-Syn aggregates. It has been proposed that the intermediate aggregates including oligomers and protofibrillar are toxic to cells through their interaction with cellular membranes and the disruption of membrane integrity [1, 2]. Furthermore, it has been shown that *α*-Syn aggregates could be transported between neurons and act as seeds, thereby propagating synuclein pathology [3, 4].

More than 90% of the cases of PD are sporadic with no familial background [5]. Despite the wide range of studies in this field, little is known regarding the causes of *α*-Syn misfolding and aggregation in the brain. Given that no certain preventive therapy is available against *α*-Syn fibrillation and its dangerous effects, it would be better to avoid factors which may facilitate the fibrillation phenomenon. Strong evidence shows that there is a relationship between exogenous materials and proteins misfolding as well as amyloid fibrillation. In vitro studies indicate that the variety of materials including metal ions (e.g., Ca, Mg, Zn, and Fe), drugs, and small molecules, as well as nanoparticles, can modulate the *α*-Syn aggregation rates [5–8]. It has also been demonstrated that toxins can intensify *α*-Syn aggregation leading to the PD pathology. Regardless of the ability to easily access all parts of the body, the candidate toxins are assumed not to have access to the brain because of BBB. However, toxins can still enter the brain through olfactory, gastrointestinal tract, and the vagus nerve, where toxins migrate and are transported [9, 10]. There is a direct link between the central nervous system (CNS) and nasal crater where toxins are capable of reaching the brain through the intranasal application; for instance, after intranasal administration of 1-methyl-4-phenyl-1,2,3,6-tetrahydropyridine (MPTP) it induced a dramatic degeneration in the substantia nigra [11, 12]. Additionally, high rates of PD have been reported in farmers and also household members exposed to herbicides and pesticides [13, 14]. As a result of the neural connection between the nasal crater and the brain, a new strategy of drug delivery to the CNS is through the nasal delivery system, a noninvasive conduit especially for the treatment of neurodegenerative diseases such as Alzheimer's disease (AD) and PD [15–17]. On the other hand, there are many unapproved reports regarding aromatherapy against AD and PD [9, 18, 19]. The gastrointestinal tract (GI) is also considered an open accessible way to the brain. According to a new report, GI might be the start point for PD. Risk of PD has been shown to drop significantly in patients that have had the vagus nerves of their GI tract removed [20].

Medicinal plants are used traditionally to cure diseases. There is a common belief that the extracts of medicinal plants including essential oils are less dangerous than chemical medicines. Essential oils are versatile and used as flavors and fragrances that strongly stimulate the senses of taste (gustatory) and smell (olfactory) [21, 22]. Every year, large amounts of essential oils are used in dietary supplements, as food additives, or even as air fresheners [22]. Fundamentally, each essential oil includes many different complex compounds which may have different effects on health and remediation.

There are standard toxicological methods used in evaluating the toxicity and mutagenicity effects of essential oils, for instance, oral and dermal toxicity in laboratory animals or mutagenic in spore Rec assay or Ames test [23–25]. However, their effects on the amyloidogenic proteins have not been considered. It is possible that certain types of essential oils may contain compounds with special activity on particular proteins, consequently leading to amyloidosis fibrillation. Given the permeability of essential oils from nasal and gastrointestinal tract to the brain, the effects of such oils on enhancing or alleviating the trend of amyloidosis should be considered.

The purpose of this study was to discuss and determine the effects of 15 well-known Iranian essential oils on the amyloidal fibrillation of human *α*-Syn. They are generally used as flavor or fragrance additives but are also used in traditional medicine as remediation. Through using various methods, this study indicated that some of the examined essential oils, specifically* M. communis*, dramatically increased the rate of *α*-Syn fibrillation with significant effect on the nucleation phase. This effect was not detected in the fibrillation of other well-known amyloidogenic proteins, hen egg white lysozyme (HEWL), or insulin.

## 2. Material and Methods

Thioflavin T (ThT), 3-(4,5-dimethylthiazol-2-yl)-2,5-diphenyltetrazolium bromide (MTT), *α*-pinene, limonene, 1,8-cineole, linalool, 1,2-dioleoyl-sn-3-phosphatidylglycerol (DOPG), HEWL, and human insulin were obtained from Sigma-Aldrich (USA). All essential oils are purchased from Barij Essence Pharmaceutical Co. (Kashan, Iran). SH-SY5Y cell line, a subclone of human neuroblastoma SK-N-SH, was acquired from Pasteur Institute (Tehran, Iran). All salts and organic solvents were obtained from Merck (Darmstadt, Germany). The cell culture medium (DMEM) and antibiotics (penicillin, streptomycin) were purchased from GibcoBRL (Life Technologies, Paisley, Scotland). Fetal bovine serum (FBS) was obtained from Biosera (England).

### 2.1. Expression and Purification of *α*-Syn

The pNIC28-Bsa4 plasmid containing human *α*-Syn cDNA was transformed into* Escherichia coli *BL21 (DE3) pLysS (Novagen, Madison, WI, USA). Expression was induced with IPTG (1 mM) and the proteins were extracted and purified according to methods explained in [7, 26]. Briefly, after centrifugation (3500 rpm, 4°C, and 20 min), the pellet from 1 L culture was resuspended in 100 mL osmotic shock buffer (30 mM Tris-HCl, 40%, sucrose, and 2 mM EDTA, pH 8) and incubated for 10 min followed by centrifugation (9000 g, 20°C, and 30 min). The resulting pellet was resuspended in 90 mL ice-cold deionized water with 40 *μ*L of saturated MgCl_2_. The supernatant was collected after centrifugation (9000 g, 4°C, and 20 min). The recombinant protein was then purified by anion-exchange chromatography and size exclusion chromatography. The fractions were analyzed using SDS-PAGE and the collected purified *α*-Syn was dialyzed exhaustively against deionized water. The purified protein was freeze-dried and stored at −20°C.

### 2.2. Preparation of the *α*-Syn, HEWL, and Insulin Fibrils

70 *μ*M of freeze-dried human recombinant *α*-Syn was dissolved in 30 mM tris buffer (pH: 7.2) and incubated in a shaker incubator (250 rpm) with 3 mm glass beads at 37°C. For HEWL and human insulin, the buffer was 30 mM glycine (pH: 2.5). Both of these proteins were incubated in a shaker incubator (250 rpm) with 3 mm glass beads at 57°C. All buffers were supplemented with 1 mM EDTA and 0.05 mM NaNO_3_.

### 2.3. ThT Fluorescence Assay

The fibrillation process was monitored by ThT fluorescence intensity. In brief, 10 *μ*L of each sample was added to 490 *μ*L of 12 *μ*M working solution of ThT in 10 mM tris buffer (pH 8.0). Fluorescence emission spectra were then taken using excitation at 440 nm and recording the emission between 450 and 550 nm. The excitation and emission slit widths were set to 5 and 10 nm, respectively. All fluorescence experiments were carried out on a Varian Cary Eclipse fluorescence spectrophotometer (Mulgrave, Australia) at room temperature. To the normalized ThT fibrillation data and analysis, the parameters of the kinetic of fibrillation of the Finke-Watzky (F-W) two-step model [27] were fitted:

(1) Ft=11+e−4νt−t1/2,

(2) tN=t1/2−12ν,

where *t*_1/2_ is the time required to produce half the total product, *ν* is the rate of growth at that time, and *t*_*N*_ is the duration of the nucleation phase.

### 2.4. Circular Dichroism Analysis

Circular Dichroism (CD) spectra in the far-UV region (190–260 nm) were obtained on an AVIV 215 spectropolarimeter (Aviv Associates, Lakewood, NJ, USA), in 0.1 cm circular cuvettes at room temperature. The protein concentration was 0.1 mg/mL. To avoid perturbation of small aggregated particles, the sample of monomer was centrifuged at 15000 rpm for 15 minutes. However, for the incubated samples, no centrifugation was carried out, resulting in scattering and small deviates in base line.

### 2.5. Atomic Force Microscopy

The proteins incubated in the condition for fibril formation as discussed in methods were diluted 20 times in 30 mM tris buffer (pH 7.2), and small aliquots (10 *μ*L) were deposited on freshly cleaved mica sheets. Then, samples were dried using nitrogen gas, and atomic force microscopy (AFM) was performed at room temperature by a NanoScope IIId controller from Veeco Instruments Co. (Plainview, NY, USA) with a silicon probe (CP). Imaging was performed under tapping mode.

### 2.6. Preparation of Large Unilamellar Vesicles (LUVs)

Freeze-and-thaw procedure was employed for the formulation of LUVs. 1,2-Dioleoyl-sn-3-phosphatidylglycerol (DOPG) was dissolved at 5 mg/mL in PBS in the presence of calcein at self-quenching concentration (70 mM). The solution was subjected to 10 freeze-and-thaw cycles between −196°C (in the liquid nitrogen) and +50°C (in a water bath). The lipid solution was extruded 21 times through a 100 nm filter using the mini extruder (Instruchemie, Netherlands). After extrusion, vesicles solution was run on a PD-10 desalting column (GE Healthcare) to separate the free calcein from calcein entrapped vesicles.

### 2.7. Calcein Release Assay

According to an increase in the permeabilization of vesicles in the presence of the toxic aggregated species including oligomers and protofibrils, release of calcein from liposomes and the subsequent increase in the fluorescence signal due to dilution were measured. Before adding the protein, the signal of DOPG vesicles (42 *μ*M) was measured at the excitation of 485 nm and emission of 520 (considered as *A* in (3)). Then 5% (v/v) of the protein was added and the samples were incubated for 10 minutes and the fluorescence was measured again (considered as *B* in (3)). Finally, 1% (v/v) triton ×100 was added to measure the saturated end-level of fluorescence (considered as *C* in (3)). To illustrate the percentage of release, the following formula was employed:

(3) $$\begin{array}{c} \%\text{ of calcein release }=\left[\;\frac{\left(B-A\right)}{\left(C-A\right)}\;\right]*100. \end{array}$$

### 2.8. Cell Culture

The SH-SY5Y neuroblastoma cells were cultured in DMEM (high glucose) F-12K enriched with 10% fetal bovine serum, penicillin (100 U/mL), and streptomycin (100 *μ*g/mL) with a pH of ~7.2–7.4 in 5% CO_2_ humidified incubator at 37°C (all reagents from GIBCO, Invitrogen, Carlsbad, CA, USA, unless otherwise stated). The medium was changed every 2-3 days. Cells were plated with densities of 10000 cells/cm^2^ cultured. For differentiation, after 48 hours the FBS concentration was reduced to 1% while adding 10 *μ*M of retinoic acid. After 48-hour incubation in the presence of retinoic acid, 10% (v/v) incubated *α*-Syn was added to cultures.

### 2.9. MTT Assay

The MTT assay was performed for cell viability evaluations. SH-SY5Y cells (at a density of 10 × 10^4^ cells/mL) were seeded in 200 *μ*L of growth medium in a 96-well microtiter plate. After additional 48 hours of incubation with protein in proper conditions, a 20 *μ*L aliquot of MTT stock solution (5 mg/mL in PBS) was added to each well and the plates were incubated for 4 hours. Crystals of formazan were dissolved in DMSO and cell viability was measured at 570 nm by an ELISA reader (Expert 96, AsysHitch, Ec Austria). The percentage of the cell viability was calculated using (2) as follows:

(4) $$\begin{array}{c} \frac{\text{The number of live cells}}{\text{The number of total cells (live and dead cells)}}\times 100. \end{array}$$

### 2.10. Statistical Analysis

All experiments were carried out in triplicate, and data was presented as means ± SD. The collected data was analyzed using the SPSS software. Statistical significance between two groups was concluded by the unpaired Student* t*-test. Also, one-way ANOVA was employed for the results of more than two experimental groups to state differences between groups. *P* values < 0.05 were considered statistically significant.

## 3. Results and Discussion

### 3.1. Inductive Effects of Essential Oils on *α*-Syn Fibrillation

**Figure 1:**
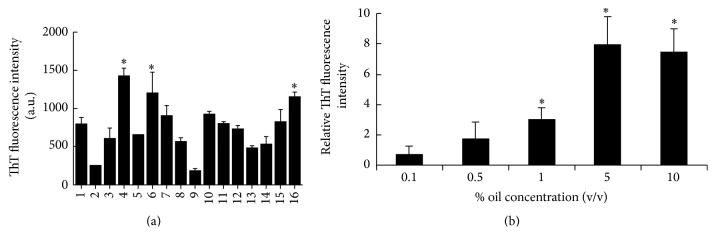
Effects of essential oils on the fibrillation of *α*-Syn assessing with ThT fluorescence assay at 482 nm wavelength. (a) *α*-Syn (70 *µ*M) was incubated at 37°C, pH 7.4, for 24 hours either (1) alone or in the presence of 1% (v/v) of (2)* Ferula gummosa*, (3)* Eucalyptus globulus*, (4)* Myrtus communis* (Myrtle), (5)* Satureja hortensis*, (6)* Rosmarinus officinalis* (Rosemary), (7)* Mentha spicata* (Spearmint), (8)* Mentha piperita* (Peppermint), (9)* Cuminum cyminum* (Cumin), (10)* Artemisia dracunculus* (Tarragon), (11)* Citrus sinensis* (Orange), (12)* Citrus limonum *(Lemon), (13)* Thymus vulgaris* (Thyme), (14)* Lavandula officinalis* (Lavender), (15)* Mentha pulegium *(Pennyroyal), and (16)* Foeniculum vulgare* (Fennel). (b) *α*-Syn (70 *µ*M) was incubated as same as (a) in the presence of different concentrations of* Myrtus communis.* Each value represents the mean ± SD (*n* = 3). ^*∗*^ *P* < 0.05 (one-way ANOVA) indicated the samples are significantly different from the control.

In this study, the effects of 15 well-known essential oils, including* Ferula gummosa, Eucalyptus globulus, Myrtus communis *(Myrtle),* Satureja hortensis, Rosmarinus officinalis *(Rosemary),* Mentha spicata *(Spearmint),* Mentha piperita *(Peppermint),* Cuminum cyminum *(Cumin),* Artemisia dracunculus *(Tarragon),* Citrus sinensis *(Orange),* Citrus limonum* (Lemon),* Thymus vulgaris *(Thyme),* Lavandula officinalis *(Lavender),* Mentha pulegium *(Pennyroyal), and* Foeniculum vulgare *(Fennel), on the fibrillation of *α*-Syn, were investigated. All of them are routinely used as food additives, flavors, and fresheners and also in traditional medicine. To explore the effect of the oils on *α*-Syn, aqueous solution of *α*-Syn (70 *μ*M) was incubated under the condition for inducing fibril formation with 1% of each oil and the fibrillation was monitored by ThT method.  Figure 1 shows that, among the tested oils,* Cumin* and* Ferula gummosa* decreased the rate of fibrillation. In another study, we showed that the main compound of* Cumin*, cuminaldehyde as an active aldehyde, can modulate the fibrillation of *α*-Syn [28]. Regarding the antifibrillation effects of* Ferula gummosa*, further studies need to be conducted. On the other hand, among other oils, three of them induced fibrillation, specifically,* M. communis*.* M. communis* oil is well known as safe oil which can be used in cosmetic and medical applications. In Iran, this oil is known as a holy plant and has been used as a traditional herb for diarrhea, hemorrhoids, and oral aphtha [29]. On the other hand, there are many studies about its beneficial use in other medical applications such as against the development of granuloma and TNF-*α* and IL-6 production [30] or as analgesic and antibacterial [31]. However, here we focus on the details of its activity on *α*-Syn fibrillation and cytotoxicity. *α*-Syn was incubated with varying concentrations of* M. communis* oil (0.1, 0.5, 1, 5, and 10% v/v).* M. communis* oil induced the *α*-Syn fibrillation in a concentration dependent manner as indicated by the ThT assay (Figure 1(b)). In the presence of up to 1% of* M. communis* oil, the *α*-Syn fibrillation was significantly increased.

### 3.2. Impact of* M. communis* Oil on the Lag Phase and Growth Rate of *α*-Syn Fibrillation

**Figure 2:**
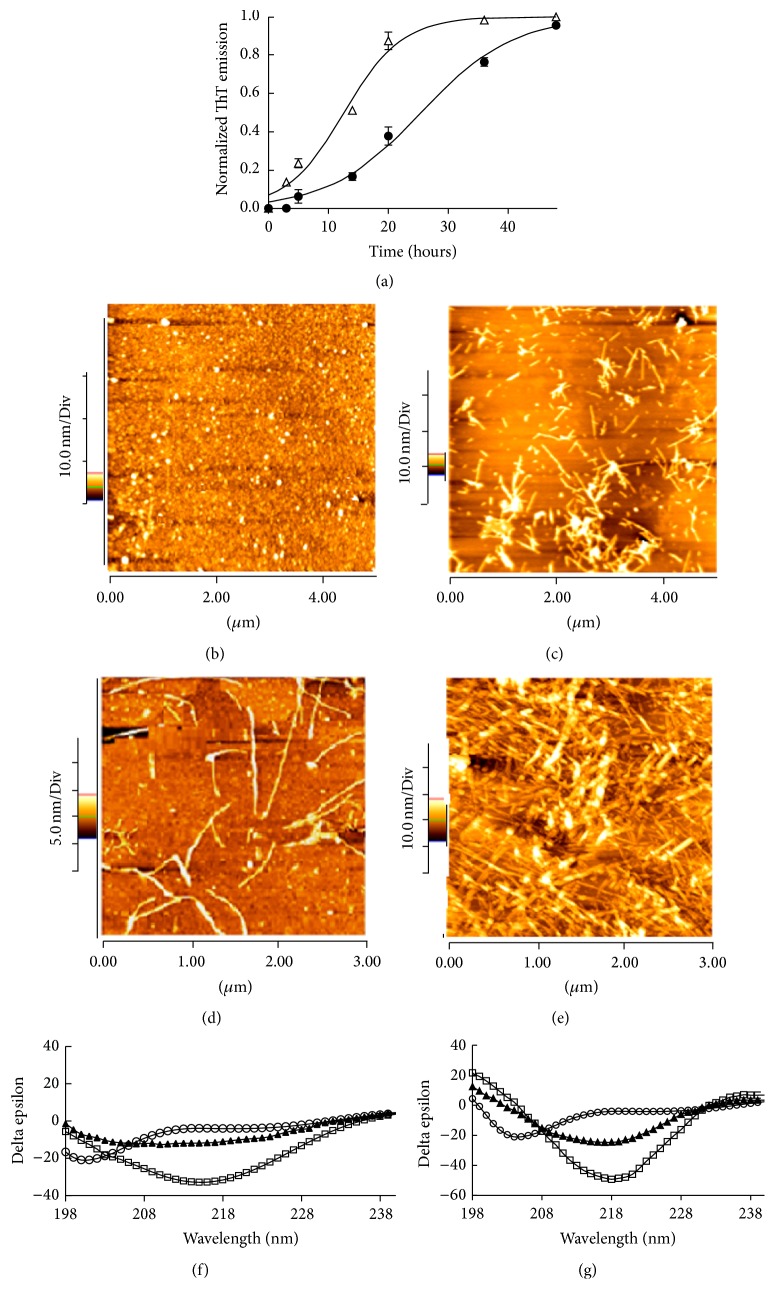
Effect of* M. communis* on the fibrillation of *α*-Syn. (a) The kinetic of *α*-Syn fibrillation in the absence (●) and the presence of 1% of* M. communis *(▵) monitored by ThT fluorescence. The continuous lines represent fits to (1). (b)–(e) AFM images and (f)-(g) far-UV CD spectra of *α*-Syn incubated without or with* M. communis *(1% v/v). (b), (c), and (f) are for *α*-Syn incubated without* M. communis*; (d), (e), and (g) are for samples incubated with* M. communis*. (▲) is considered for 7 h and (□) for 24 h incubation. (○) is reflected for fresh monomer protein. The concentration of *α*-Syn was 70 *μ*M.

The activity of* M. communis *on the kinetic of fibrillation was monitored by ThT assay (Figure 2(a)). The curves were fitted and the parameters of fibrillation were quantized using the Finke-Watzky equation [32], as described in Table 1. The process of protein fibrillation was divided into three steps which were determined from a series of complex structural transitions from monomeric *α*-Syn to oligomeric forms, and is presumably considered as neurotoxic forms and finally mature fibrils. In the early stage of *α*-Syn fibrillation, usually, some moderate changes in the structure or physical parameters of the monomeric form occurred; thus monomers are able to assemble to form oligomers. This is the nucleation or lag phase and is known as the rate limiting step of fibrillation. As shown in Figure 2(a) and Table 1,* M. communis* affected the lag phase and sharply reduced it from 9.87 to 3.97 h. It is believed that proteins and peptides in physiological condition are often thermodynamically stable in their native forms. Factors that can perturb the stability of proteins or peptides and drive them to aggregation may play important roles in amyloidosis and degeneration. After passing through the lag phase, oligomeric species usually gather quickly; in this stage, the aggregated species which mostly contain beta structures tend to bind to ThT. In the presence of* M. communis*, the rate of fibril growth (*ν*) also increased, indicating that the protein proceeds to the classic pathway of fibrillation. Although many factors are responsible for protein instability and induce aggregation progress, they induce amorphous aggregated particles in proteins [33, 34]. Studies on AFM and CD also support this assertion. The aggregated species and their dispersity for the treatment of *α*-Syn with* M. communis *are different from the untreated protein. AFM image (Figure 2(b)) indicates that 7 h aged untreated *α*-Syn contains small rounded particles, while a lot of determined fibrils appeared in 7 h aged treated *α*-Syn with* M. communis *(Figure 2(d)). The differences between treated and untreated *α*-Syn are clearer in the 24 h aged incubated protein, which is the sample treated with* M. communis*, and produced a high level of expanded fibrils (Figure 2(e)) compared to the control (Figure 2(c)). Far-UV CD of *α*-Syn monomer gives a strong negative peak around 200 nm; the appearance of this peak is due to the random coil structure of the protein. During the formation of the fibrils, *α*-Syn forms cross-*β*-sheets, causing a negative absorption of light at a wavelength of 218 nm. Interestingly, a strong negative peak at a wavelength of 218 nm was detected when *α*-Syn was incubated for 7 h with* M. communis *(Figure 2(g)). However, this peak was not detected in untreated *α*-Syn after 7 h of incubation (Figure 2(f)). Furthermore, despite the fact that either untreated or treated *α*-Syn with* M. communis *after 24 h of incubation contained high amounts of beta strand structures, it revealed that the treated protein comprised more beta sheet structure than the untreated *α*-Syn, with a sharper negative peak around 218 nm.

### 3.3. *M. communis* Oil Has an Increasing Role on *α*-Syn Membrane-Permeability and Cytotoxicity

**Figure 3:**
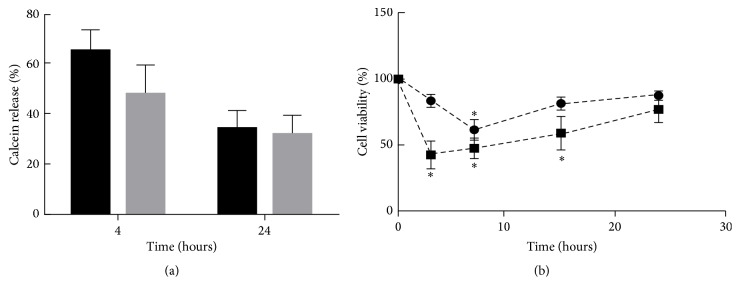
The effect of* M. communis* on the permeability and toxicity of *α*-Syn. *α*-Syn was incubated for different times in the presence (■, black) and absence (●, gray) of* M. communis* (1% v/v). (a) The calcein release from DOPG vesicles after adding *α*-Syn aggregates formed over 4 and 24 h. (b) The viability of the SH-SY5Y cells after 48 h incubation with *α*-Syn aggregated forms over different time (0–24 h). Each value represents the mean ± SD (*n* = 3). ^*∗*^ *P* < 0.05 (Student's* t*-test) indicated the samples are significantly different from the control. Controls are the viability of the SH-SY5Y cells treated with buffers.

Evidences from different analytical approaches indicate that there is a strong relationship between membrane perturbation and the level of toxic aggregated species of *α*-Syn. To verify the effect of* M. communis *on *α*-Syn membrane-permeability, the rate of calcein release from a unilamellar vesicle was assessed (Figure 3(a)). The amount of calcein in the DOPG vesicles was high, resulting in an autoquenching of its fluorescence. When calcein is released into the solution and diluted, the intensity of its fluorescence is enhanced. As indicated in Figure 3(a), the release of calcein was increased (40–60%) when DOPG vesicles were exposed to *α*-Syn. The rate of release in the samples which were treated with the oil was higher than the untreated ones and even higher during the initial time (4 h). Adding the oil alone to the solution did not show any increase in the fluorescence intensity (data not shown), indicating that the membrane-permeability of *α*-Syn treated with* M. communis *may berelated to the aggregated protein.

To evaluate the toxicity of *α*-Syn during the fibrillation process in the absence and presence of* M. communis*, samples were taken at different time intervals of 0, 3, 7, 15, and 24 h. The cultured SH-SY5Y cells were treated with 10% (v/v) of resulting samples and the viability was monitored using MTT assay. In cases where the cells were exposed to untreated protein, the toxic effects progressed up to 7 h and then decreased (Figure 3(b)). The cytotoxic effect of the protein in the presence of* M. communis *showed differences in cell viability rate and the time of the uppermost toxicity, suggesting that* M. communis *caused the production of the aggregated species with profound higher toxic effects than the aggregates formed in the absence of oil, especially in the short periods of incubation. As shown in Supplementary Figure 1 (see Supplementary Material available online at http://dx.doi.org/10.1155/2016/6219249), the morphology cells were changed in the treated cultures after 48 hours of incubation.

Higher cytotoxicity of the aggregates which were generated in the presence of* M. communis *may be due to the induction and formation of a high amount of toxic oligomers in a short period of time. It has been shown that the different aggregated species formed during fibrillation have different levels of cytotoxicity with different mechanisms [35]. The most cytotoxic forms may be oligomers and protofibrils with a high tendency to perturb the stability and homogeneity of biological membranes. In this study, ThT fluorescence assay, as well as AFM images, showed that, in the presence of* M. communis*, the velocity of fibrillation and also its intensity were increased. These data suggest that when the oil was used with *α*-Syn, high toxic aggregated species were formed earlier and at a higher level.

### 3.4. Reduction of the Inhibitory Effects of Baicalein and Cuminaldehyde on *α*-Syn Fibrillation in the Presence of* M. communis*

**Figure 4:**
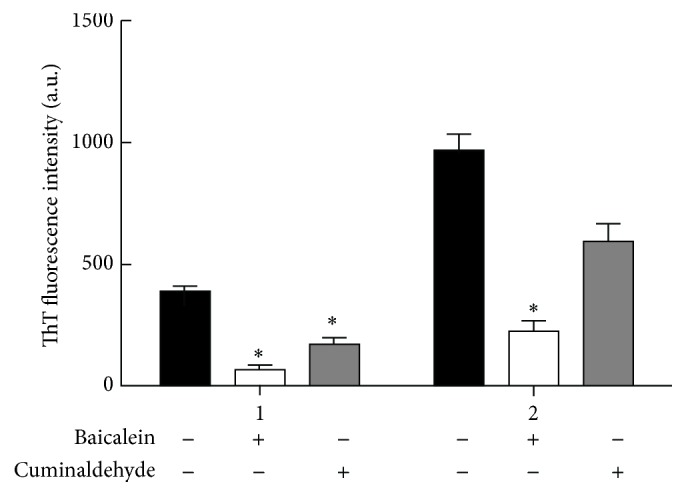
Effect of* M. communis* on the inhibitors of *α*-Syn fibrillation. The rate of *α*-Syn fibrillation with 400 *μ*M baicalein (white) and cuminaldehyde (gray) was monitored by ThT fluorescence. There is no* M. communis* in series (1) and there is 1% (v/v) of* M. communis* in series (2). The black columns represent *α*-Syn with no inhibitors. Each value represents the mean ± SD (*n* = 3). ^*∗*^ *P* < 0.05 (one-way ANOVA) indicated the samples are significantly different from the control.

It has been shown that aggregated *α*-Syn tends to propagate between synaptically connected neurons and exosomal release and has unveiled a new therapeutic target for PD. In this direction, different groups have performed the screening of compounds to detect the ones which inhibit *α*-Syn fibrillation and oligomerization. Baicalein and cuminaldehyde are both known as *α*-Syn fibril inhibitors and are naturally found in medicinal plants. It is assumed that their inhibitory effects persist in the presence of a fibril inducer such as* M. communis. *As shown in Figure 4, in the presence of* M. communis*, the level of fibrillation increased dramatically especially for the proteins treated with cuminaldehyde. Baicalein mostly influences the oligomerization step by changing the polymerization kinetic and inducing the off-pathway of oligomers production [28]. However, cuminaldehyde mostly affects the nucleation step and monomeric form of protein by modification of lysine residues [28]. This data suggests that* M. communis *activity begins from early stages of the fibrillation, which is similar to the kinetic study (Figure 2 and Table 1) where the *t*_*N*_ rate was considerably changed. On the other hand, the strength of its effect is much higher than the neutral inhibitors' effects and can overcome their influence and activity.

### 3.5. The Major Compounds Present in* M. communis* Oil Do Not Enhance *α*-Syn Fibrillation and Also* M. communis* Oil Does Not Enhance the Fibrillation in HEWL and Insulin

**Figure 5:**
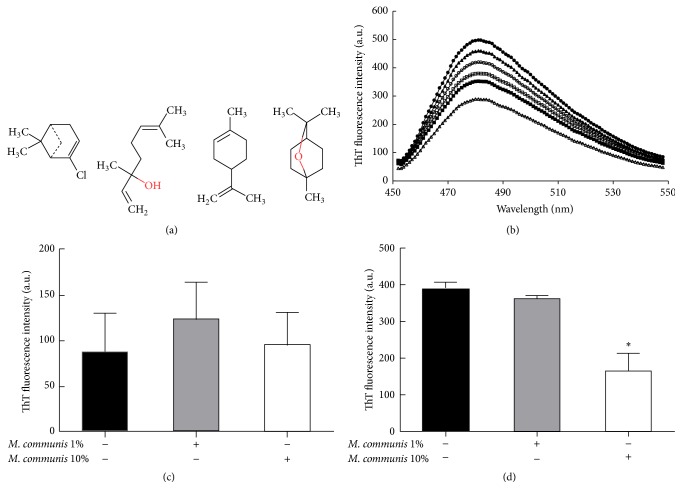
(a) Chemical structures of *α*-pinene, limonene, 1,8-cineole, and linalool (left to right, resp.). (b) The *α*-Syn fibrillation rate in the absence (●) and presence of 2% (v/v) of *α*-pinene (■), limonene (∆), 1,8-cineole (▲), linalool (○), and the heated oil (□). (c) The fibrillation rate of 2 mg/mL of insulin and (d) HEWL in the absence (black) and the presence of 1% (gray) and 10% (white) (v/v) of* M. communis*. The proteins were diluted in glycine buffer (30 mM; pH 2.5) and treated with the oil and then incubated for 24 hours at 57°C. The fibrillation rate was measured by ThT fluorescence assay. The chemical structures were drawn by Marvin-Sketch software. ^*∗*^ *P* < 0.05 (one-way ANOVA) indicated the samples are significantly different from the control.

A GC-mass analysis was performed for the* M. communis *oil and its data is presented in Supplementary Table 1. GC-mass spectrometry detected 36 compounds in the* M. communis* oil with very different chemical structures. The four compounds which had more occurrence of the selected oil are *α*-pinene, limonene, 1,8-cineole, and linalool. In another study on the evaluation of compounds presented in the* M. communis* oil obtained from Iran, using GC-mass spectrometry method, the same results have been achieved [36]. Their chemical structures are different as presented in Figure 5(a). These, and other compounds which are present in* M. communis *oil, have reactive groups which may influence the fibrillation process by reacting with *α*-Syn. In this study, the influence of the four mentioned compounds was assessed on the *α*-Syn fibrillation, by monitoring the ThT fluorescence intensity after 24 h incubation (Figure 5(b)). The presence of *α*-pinene, 1,8-cineole, and limonene with *α*-Syn during the fibrillation processes was shown not to enhance the outcome but rather inhibited the effect on fibrillation.

Subsequently, the oil was incubated in open door at 37°C for 4 h and the prewarmed oil was added to the protein. Unexpectedly, addition of 1% of the prewarmed oil triggered a significant decrease of the fibrillation as presented in Figure 5(b). It is implicit that after heating, some more versatile compounds evaporate. On the other hand, it seems that active compounds impact *α*-Syn immediately after addition. The GC-mass analysis also indicated that after heating, the composition of* M. communis* oil was changed (Supplementary Table 2). Recently, it was shown that the compounds which are present in the essential oils have controversial effects on the fibrillation process. For example, limonene and terpinolene, which are present in* Cuminum cyminum *essential oil, indicated opposite effects on the HEWL fibril formation [37]. Therefore, as a result of the complexity of the composition of the oils, it is extremely difficult to find the real mechanism and a precise compound of the oils which influence the fibrinogenic proteins. It is possible that some compounds have synergistic or antagonistic impacts on each other. On the other hand, the oils are usually used as crude extracts, so it is better to verify their whole extract effects.

Next, to identify the considerable activity of* M. communis *on the fibrillation and cytotoxicity of *α*-Syn, the oil's effects on two other well-known amyloidogenic proteins were examined. Human insulin is a small protein which is believed to form local fibrillar structures in the distal and proximal places of the injection site in diabetic patients [38]. Its fibrillar potency has been studied well and used as a suitable model with the other well-known protein, HEWL, in the studies related to fibrillation process. After adding* M. communis *(1 and 10% (v/v)) to either insulin or HEWL samples, the rate of fibril formation was assessed after 24 h incubation under fibrillation condition (pH: 2.5 and 57°C). As shown in Figure 5(c), treatment of insulin with the oil did not affect fibrillation. However, results showed that* M. communis *did not always have enhancing effects on the fibril formation of proteins but may have inhibitory effects as determined for HEWL fibrillation in a concentration dependent manner (Figure 5(d)). Given this observation, it can be concluded that* M. communis *contains various compounds which may have different effects on dissimilar peptides and proteins. These differences depend on the chemical and physical properties of proteins, as well as the compositions of oils.

## 4. Conclusion

A growing concern regarding the prevalence of neurodegenerative diseases in the world has arisen due to the incurable nature of these diseases, increasing patient population and its socioeconomic feedback. One aspect that could be considered is the reduction of risk factors. Considering that protein fibrillation plays an indisputable role in the development and progress of these diseases, factors that induce or accelerate the fibrillation process can be considered as risk factors. Considering that the nose and gut have routes to the brain, the effect of some essential oils, which are used in the herbal medicines and aroma industries and have the ability to pass through the brain barrier, was investigated. In the present study, it was shown that three essential oils including* M. communis*,* Foeniculum vulgare*, and* Rosmarinus officinalis* had a resonator effect on the fibrillation of *α*-Syn and among them* M. communis *had the greatest impact. This result was influenced by both the lag phase and the growth phase of fibrillation. The generated aggregates were also more toxic in the presence of the essential oil. Although this essential oil had a significant impact on fibrillation, this study showed that the components, which constitute a major contribution of this essential oil, are not the cause of this change, suggesting the complexity of the effects of the extract and the synergetic effects of the present compounds in oil, even in small amounts. In addition, this effect on the other two fibrinogenic proteins, insulin and HEWL, was not observed, indicating that such impacts depend on the characteristics of the protein. One point that is noteworthy is the use of this essential oil and other extracts of* M. communis* worldwide and especially in some religious ceremonies. Zoroastrians particularly use this plant during religious ceremonies, and there are reports that the incidence of PD is relatively high among this group. For consumable materials, which are likely to pass through the brain such as volatile compounds, this kind of experiment can be considered on proteins and peptides involved in the NDs. Ways of treating amyloidosis ND may not be easy, nor within reach, but it is important to improve prevention and care methods.

## Supplementary Material

As shown in Supplementary Figure 1 SH-SY5Y cells cultured which were treated with 5% 7 h incubated *α*-Syn have very different morphology and confluency in comparison with those cultured under no treating. While the effect of *α*-Syn on SH-SY5Y cells is attributed to formation of toxic aggregated species of *α*-Syn, it seems that *M. communis* caused increase of the toxic aggregated species of the *α*-Syn. Supplementary Table 1 and Table 2 show the main compounds of *M. communis* oils before and after heating, respectively. After heating, the effects of *M. communis* on the fibrillation and toxicity of *α*-Syn became reduced.

## Figures and Tables

**Table 1:** Kinetic parameters of *α*-Syn fibrillation as a function of 1% of *M. communis *calculated with (1).

	*ν*	*t*_1/2_	*t*_*N*_
*α*-Syn	0.033	13.536	9.87
+*M. communis*	0.053	25.214	3.97
Relative value	1.602	0.53	0.405

The relative values are represented as relative to values in the absence of *M. communis*: relative growth rate (*ν*/*ν* control), relative half-time (*t*_1/2_/*t*_1/2_ control), and relative lag time (*t*_*N*_/*t*_*N*_ control).
