# Supplementary material for: Essential Oils May Lead α-Synuclein towards Toxic Fibrils Formation
Source: Parkinsons Dis. 2016 May 24;2016:6219249. doi: 10.1155/2016/6219249 (PMC4894988; doi:10.1155/2016/6219249)
Supplement: Supplementary file 1 — As shown in Supplementary Figure 1 SH-SY5Y cells cultured which were treated with 5% 7 h incubated α-Syn have very different morphology and confluency in comparison with those cultured under no treating. While the effect of α-Syn on SH-SY5Y cells is attributed to formation of toxic aggregated species of α-Syn, it seems that M. communis caused increase of the toxic aggregated species of the α-Syn. Supplementary Table 1 and Table 2 show the main compounds of M. communis oils before and after heating, respectively. After heating, the effects of M. communis on the fibrillation and toxicity of α-Syn became reduced. [file 6219249.f1.docx]

Supplementary discussion: as shown in Supplementary Figure 1 SH-SY5Y cultured witch treated with 5% 7h-incubated α-Syn have very different morphology and confluency in comparison with the cultured under no treating. While the effect of α-Syn on SH-SY5Y cells is attributed to formation of toxic aggregated species of α-Syn, it seems that *M. communis* caused to increase the toxic aggregated species of the α-Syn. Supplementary Table 1 and table 2 show the main compounds of M. communis oils before and after heating, respectively. After heating, the effects of *M. communis* on the fibrillation and toxicity of α-Syn became reduced.


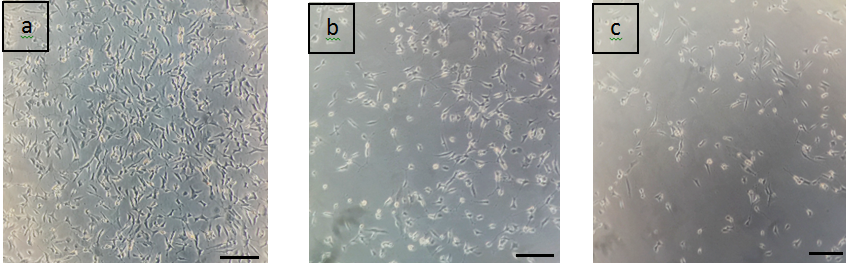


a

c

b

Supplementary Figure 1: Morphological analysis of SH-SY5Y cultured for 48 hours in the absence (a) and presence of 5% 7h-incubated α-Syn without(b) and with *M. communis* (1% (v/v)). 20X magnification, Scale bar=100µm

Supplementary Table 1: Chemical composition of the *Myrtus communis’* essential oil

| No. | Compound | RT | % |
| --- | --- | --- | --- |
| 1 | Propionic acid | 6.6 | 0.1 |
| 2 | α-Pinene | 6.99 | 35.1 |
| 3 | Camphene | 7.85 | 0.6 |
| 4 | β-Pinene | 8.62 | 1.45 |
| 5 | β-Myrcene | 8.95 | 0.32 |
| 6 | 1-Phellandrene | 9.45 | 0.14 |
| 7 | 3-Carene | 9.63 | 0.42 |
| 8 | Limonene | 10.15 | 21.5 |
| 9 | 1,8-Cineole | 10.95 | 18.5 |
| 10 | Cis-Ocimene | 11.11 | tr |
| 11 | **γ-**Terpinene | 11.5 | 0.2 |
| 12 | Linalool | 12.04 | 8.21 |
| 13 | Endo-Fenchol | 13.8 | tr |
| 14 | Terpinene-1-ol | 14.59 | 0.13 |
| 15 | Trans-Pinocarveol | 14.85 | tr |
| 16 | Camphor | 15.2 | 0.25 |
| 17 | Bomeol | 15.57 | 0.63 |
| 18 | 3-Cyclohexen-1-ol | 16.35 | 0.44 |
| 19 | α-Terpineol | 17.18 | 2.45 |
| 20 | γ-Terpineol | 17.33 | 0.33 |
| 21 | Trans-Geraniol | 18.52 | 0.1 |
| 22 | Linalyl acetate | 19.62 | 2.27 |
| 23 | Geraniol | 19.78 | 0.7 |
| 24 | α-Terpinenyl acetate | 21.93 | 0.22 |
| 25 | Geranyl acetate | 24.12 | 1.45 |
| 26 | Trans-Caryophyllene | 26.25 | 0.8 |
| 27 | α-Humolene | 27.73 | 0.14 |
| 28 | Varatraldehyde | 30.24 | 0.22 |
| 29 | Caryophellene oxide | 32.25 | 0.19 |
| 30 | o-Menth-8-ene | 33.68 | tr |

Supplementary Table 2: Chemical composition of the Myrtus communis’ essential oil after heating

| No. | Compound | RT | % |
| --- | --- | --- | --- |
| 1 | α-Pinene | 7.4 | 35.1 |
| 2 | Camphene | 7.95 | 1.6 |
| 3 | β-Pinene | 8.74 | 3.45 |
| 4 | β-Myrcene | 8.95 | 0.52 |
| 5 | Limonene | 10.95 | 21.5 |
| 6 | 1,8-Cineole | 11.25 | 28.5 |
| 7 | **γ-**Terpinene | 11.81 | 1.85 |
| 8 | Linalool | 13.04 | 4.21 |
| 9 | Camphor | 15.2 | 1.85 |
| 10 | Bomeol | 15.82 | 1.63 |
| 11 | 3-Cyclohexen-1-ol | 16.55 | 0.84 |
| 12 | α-Terpineol | 17.28 | 1.45 |
| 13 | γ-Terpineol | 17.43 | 0.33 |
| 14 | Trans-Geraniol | 18.72 | 0.1 |
| 15 | Linalyl acetate | 19.62 | 0.68 |
| 16 | Geraniol | 19.88 | 0.81 |
| 17 | α-Terpinenyl acetate | 22.43 | 0.32 |
| 18 | Geranyl acetate | 24.92 | 0.45 |
| 19 | Trans-Caryophyllene | 26.55 | 1.2 |
